# Supplementary material for: Reconciling Mining with the Conservation of Cave Biodiversity: A Quantitative Baseline to Help Establish Conservation Priorities
Source: PLoS One. 2016 Dec 20;11(12):e0168348. doi: 10.1371/journal.pone.0168348 (PMC5173368; doi:10.1371/journal.pone.0168348)
Supplement: S1 Dataset — (ZIP) [file pone.0168348.s002.zip › Taxa/Serra Norte/SN_2013/N4E/Lista N4E-93.pdf]

CAVIDADE N4E-0093

Classe	Ordem	Fam/Outros	Gên/Outros	Espécie	S-U	U-S	Zon.
Arachnida	Acari	Anystidae	*Erythracarus*	sp.1	X		E
Arachnida	Acari	Macrochelidae		sp.1		X	E
Arachnida	Acari	Prostigmata		sp.49	X		P
Arachnida	Acari	Rhagidiidae		sp.20	X		A
Arachnida	Acari	Trombiculidae		sp.41	X		P
Arachnida	Amblypygi	Phrynidae	*Heterophrynus*	*longicornis*	1	1	E/P
Arachnida	Araneae	Barychelidae		jovem		1	P
Arachnida	Araneae	Corinnidae		jovem		1	E
Arachnida	Araneae	Ctenidae		jovem		1	P
Arachnida	Araneae	Ochyroceratidae	*Ochyrocera*	sp.3	X		P
Arachnida	Araneae	Ochyroceratidae	*Speocera*	sp.1	X	X	P
Arachnida	Araneae	Pholcidae	*Leptopholcus*	sp.1	X	X	E/P
Arachnida	Araneae	Pholcidae	*Mesabolivar*	aff. *cambridgei*	X		E
Arachnida	Araneae	Pholcidae	*Mesabolivar*	sp.1	X	X	E/P
Arachnida	Araneae	Pholcidae	Ninetinae	sp.1		X	P
Arachnida	Araneae	Salticidae		jovem	X	X	E
Arachnida	Araneae	Scytodidae	*Scytodes*	jovem		1	E
Arachnida	Araneae	Theraphosidae	aff. *Holothele*	sp.1	1	1	P
Arachnida	Araneae	Theridiidae	*Episinus*	sp.1	X		E
Arachnida	Araneae	Theridiosomatidae	*Plato*	sp.1	X	X	E/P/A
Arachnida	Opiliones	Cosmetidae	*Roquettea*	*singularis*	2		E
Arachnida	Opiliones	Escadabiidae		sp.2	X	X	P/A
Arachnida	Pseudoscorpiones	Bochicidae		sp.1	X		A
Arachnida	Pseudoscorpiones	Chthoniidae	*Pseudochthonius*	sp.1	X	X	E/P/A
Arachnida	Schizomida	Hubbardiidae	aff. *Rowlandius*	sp.1	X	X	E/P/A
Chilopoda	Geophilomorpha	Ballophilidae	*Ityphilus*	sp.1	1	2	P/A
Chilopoda	Scolopendromorpha	Scolopendridae	*Otostigmus*	sp.1	1		P
Chilopoda	Scutigeromorpha	Pselliodidae	*Sphendononema*	*guildingii*		1	P
Diplopoda	Polydesmida	Fuhrmannodesmidae		jovem	X		P
Diplopoda	Polydesmida	Paradoxosomatidae		sp.1		1	E
Diplopoda	Polydesmida	Pyrgodesmidae		sp.1	X	X	E/P/A
Diplopoda	Spirostreptida	Pseudonannolenidae	*Pseudonannolene*	jovem	4		E/P/A
Entognatha	Collembola	Isotomidae		sp.1	X	X	E/A
Entognatha	Collembola	Paronellidae		sp.1	X		E/P
Entognatha	Collembola	Paronellidae		sp.4	X	X	P/A
Entognatha	Collembola	Sminthuroidea		sp.2	X		P/A
Entognatha	Diplura	Campodeidae		sp.1	X		E
Insecta	Blattodea	Blattidae		sp.4	6	3	E/P
Insecta	Coleoptera	Ptiliidae		jovem	X		E
Insecta	Coleoptera	Staphylinidae	Pselaphinae	sp.20	X		P
Insecta	Coleoptera	Staphylinidae	Pselaphinae	sp.35		X	E
Insecta	Coleoptera	Staphylinidae		sp.59	X		P
Insecta	Diptera	Cecidomyiidae	Cecidomyiinae	sp.	X	X	E
Insecta	Diptera	Chironomidae		sp.	X	X	E/P
Insecta	Diptera	Culicidae	*Culex*	sp.		X	P/A
Insecta	Diptera	Dolichopodidae		sp.	X		E
Insecta	Diptera	Phoridae	Metopininae	sp.		X	E
Insecta	Diptera	Psychodidae	Phlebotominae	sp.	X	X	E/P
Insecta	Diptera	Tipulidae	Tipulinae	sp.	X	X	E/P

Insecta	Heteroptera	Cydnidae		jovem	X		A
Insecta	Heteroptera	Reduviidae	Emesinae	sp.8		3	E/P
Insecta	Heteroptera	Reduviidae	Reduviinae	sp.	1	2	E/P
Insecta	Homoptera	Cixiidae		sp.3		X	E
Insecta	Hymenoptera	Bethylidae		sp.2		X	A
Insecta	Hymenoptera	Formicidae	*Camponotus*	sp.1		X	P
Insecta	Hymenoptera	Formicidae	*Camponotus*	sp.2	X	X	E
Insecta	Hymenoptera	Formicidae	*Eurhopalothrix*	sp.1	X		P
Insecta	Hymenoptera	Formicidae	*Hypoponera*	sp.1	X		E
Insecta	Hymenoptera	Formicidae	*Pachycondyla*	sp.2	X	X	E
Insecta	Hymenoptera	Formicidae	*Paratrechina*	sp.1	X		E
Insecta	Hymenoptera	Formicidae	*Paratrechina*	sp.2	X		E
Insecta	Hymenoptera	Formicidae	*Solenopsis*	sp.1	X	X	P/A
Insecta	Lepidoptera	Noctuoidea		sp.5		9	E
Insecta	Lepidoptera			jovem	X	X	E/P
Insecta	Neuroptera	Myrmeleontidae		jovem		X	E
Insecta	Orthoptera	Phalangopsidae	*Phalangopsis*	sp.		10	E/P/A
Insecta	Psocoptera	Epipsocidae	*Epipsocus*	sp.2	X		P
Insecta	Psocoptera	Manicapsocidae	*Nothoentomum*	sp.1	X		E
Insecta	Psocoptera	Psyllipsocidae	*Psyllipsocus*	sp.1	X		E
Insecta	Psocoptera			jovem	X	X	E/P
Gastropoda	Pulmonata	Bulimulidae	*Naesiotus*	sp.	X		A
Gastropoda	Pulmonata	Subulinidae		sp.4		X	A
Gastropoda	Pulmonata	Systrophiidae	*Happia*	sp.	X		E
Malacostraca	Isopoda	Armadillidae		sp.1	X		E/P
Malacostraca	Isopoda	Philosciidae		sp.1	X	X	P/A
Malacostraca	Isopoda	Philosciidae		sp.2	X		E
Symphyla		Scutigerellidae		sp.	X	X	E/P
Amphibia	Anura	Strabomantidae	*Pristimantis*	cf. *fenestratus*	2		P
Mammalia	Chiroptera	Emballonuridae	*Peropteryx*	*kappleri*		4	E/P/A
Mammalia	Chiroptera	Furipteridae	*Furipterus*	*horrens*	6		E/P/A
Mammalia	Chiroptera	Phyllostomidae	*Carollia*	*perspicillata*	1	1	P
Mammalia	Chiroptera	Phyllostomidae	*Micronycteris*	*microtis*	1		A
